# Targeting everyday decision makers in research: early career researcher and patient and public involvement and engagement collaboration in an AI-in-healthcare project

**DOI:** 10.1186/s40900-025-00753-9

**Published:** 2025-08-19

**Authors:** Duncan J. Reynolds, Susan Mountain, Victoria Bartle, Elizabeth Remfry, Michael R. Barnes, Nick J. Reynolds, Alexandra Thompson, AI MULTIPLY, Vanesa Apea, Mike Barnes, Victoria Bartle, Alastair Burt, Dexter Canoy, Megan Clinch, Ceri Durham, Olivia Grant, Soraia Guerra-Sousa, Barbara Hanratty, Tom Lawton, Hamish McAllister-Williams, Paolo Missier, Chris Plummer, Nick Reynolds, Sohan Seth, Deborah Swinglehurst, Adam Todd, Neil Watson, James Wason

**Affiliations:** 1 Wolfson Institute of Population Health, Mary University of London, London, Queen UK; 2 Patient and Public Involvement and Engagement Group, AI MULTIPLY, Newcastle, UK; 3 William Harvey Research Institute, Queen Mary University of London, London, UK; 4 NIHR Biomedical Research Centre and Department of Dermatology, Royal Victoria Infirmary, Newcastle Hospitals NHS Foundation Trust, Newcastle Upon Tyne, UK; 5 Population Health Sciences Institute Faculty of Medical Sciences, Newcastle University, Newcastle Upon Tyne, UK

**Keywords:** Patient and public involvement, Early career researchers, Artificial intelligence, Healthcare research, Ethnography

## Abstract

Patient and Public Involvement and Engagement (PPIE) is critical in the development and application of Artificial Intelligence (AI) in healthcare research to ensure that outcomes align with patients’ and the public’s needs. However, current PPIE practices often limit involvement to reactive tasks such as reviewing documents and providing plain English summaries. Whilst important, this approach can sideline PPIE from influencing key research decisions. Consequently, PPIE interactions often fail to adequately reach and influence everyday decision makers. On AI and big data research projects, these decisions are often made by Early Career Researchers (ECRs) who play a vital role in the day-to-day research process. After realising these limitations, and to address them, the NIHR-funded AI MULTIPLY consortium introduced twice-monthly "ECRs meet PPIE" sessions. These sessions began in May 2024 and enabled ECRs to present and discuss work in progress and gain targeted input from PPIE members during early phases of research, such as research direction, data and variable selection. By integrating PPIE at this stage, the project aimed to improve the relevance and impact of the healthcare research but also provide ECRs with essential skills in public engagement. At time of writing, 12 sessions have been conducted. Through ethnographic observations integrated with internal surveys, the findings show how the sessions were developed, overcame challenges, and helped to embed PPIE contributors’ voices into an AI-in-healthcare project. Based on our findings we have identified 5 recommendations for other large interdisciplinary consortia to strengthen the contribution of PPIE to everyday decision-making in research.

**Supplementary Information:**

The online version contains supplementary material available at 10.1186/s40900-025-00753-9.

## Abstract (plain language summary)

Patient and Public Involvement and Engagement (PPIE) helps make healthcare research meet patient needs, but it is sometimes limited to tasks like reviewing documents or writing plain English summaries. This can keep patients away from key decisions made early in the research process. Early Career Researchers (ECRs), who are at the start of their careers, often make important decisions like choosing which data to include, but they usually have little interaction with patients or the public. This can mean that research might not look at what patients feel is important, especially when using complicated tools like artificial intelligence (AI). To try and improve this, the NIHR-funded AI MULTIPLY project introduced twice-monthly "ECRs meet PPIE" sessions. These sessions let ECRs share their work with PPIE members early on, getting input on shaping the research direction and choosing data. This early involvement helps our research to meet patient needs. As well as this, the ECRs gained valuable skills in public engagement, communication, and professional development. Since starting in May 2024, we have had 12 sessions, with positive feedback. By observing the sessions and asking people to fill out surveys, positives and challenges of meetings were discovered. We were able to show how ECRs report improved confidence, insights, and skills, while PPIE contributors feel empowered and valued for their role in shaping research. This approach shows how PPIE can link researchers and the public, supporting patient-centred AI innovations in healthcare.

**Supplementary Information:**

The online version contains supplementary material available at 10.1186/s40900-025-00753-9.

## Background

The development and implementation of Artificial Intelligence (AI) in healthcare is advancing rapidly, with proponents of these systems promising transformative impacts. These are said to have the potential to improve diagnostic accuracy, enhance treatment efficacy, and streamline healthcare delivery [1–4], leading to better patient outcomes and more efficient use of healthcare resources. AI tools currently in use in the UK National Health Service (NHS) include C the Signs (cancer diagnosis) [5], Deep Medical (to tackle missed appointments) [6], da Vinci (AI robotic surgery assistance) [7] and many more [8]. Despite the rapid development, implementation, and potential benefits of these tools, their design often occurs in isolation from those most affected by them: patients [9, 10]. The potential risks of this exclusion are significant, as AI solutions that do not reflect patients’ needs and priorities may exacerbate health disparities or result in technologies that fail to gain trust and widespread adoption [11–17].

Patient and Public Involvement and Engagement (PPIE) offers a potential mechanism to redress these problems with AI research, development and implementation. Defined by the National Institute of Health and Care Research (NIHR) as research which is “carried out ‘with’ or ‘by’ members of the public rather than ‘to’, ‘about’ or ‘for’ them” [18, 19], PPIE has long played a role in healthcare research [20, 21], with the UK Standard for Public Involvement highlighting the importance of including the public in all stages of the research cycle [22]. In theory, PPIE ensures that patients and the public actively contribute to the research process, influencing decisions to align healthcare innovations with real-world needs [23, 24]. However, it has often been practiced in ways that are tokenistic, perpetuate power imbalances means that patient voices are ignored [25–28]. Involvement is often limited to such as only reviewing documents or providing feedback on research summaries [29–31]. While review activities are valuable, they often occur after critical research decisions have been made, relegating PPIE to a reactive rather than a proactive process. Furthermore, it often limits opportunities for PPIE engagement can take place that is not with researchers who are making everyday decisions [32, 33]. By “everyday decision makers” we refer to those individuals who are responsible for making decisions about routine daily research activities. In a large interdisciplinary consortium tacking the development of complex AI technologies in healthcare these kinds of decision might include data selection, model analysis or result representation, and are typically made by ECRs. This reactive approach sidelines the potential contributions of PPIE in shaping foundational aspects of healthcare AI development, such as the framing of research questions, data selection, and algorithm design. PPIE involvement within AI-in-health research must be proactive to help develop AI solutions that are more equitable, trustworthy, effective, and in line with the needs of patients [11].

This research aims to show how PPIE can have a positive impact on AI research by proactively engaging those making everyday decisions. Previous research has shown approaches to involving patients and the public in decision making, with strategies such as patient-centred care, self-directed care, and shared decision-making [34, 35]. However, how everyday decision makers in AI-in-healthcare research are targeted and engaged is an overlooked area of study. PPIE work often specifically aims to target senior team members [36, 37], often with good reason such as aiming to influence overall strategy and targeting those who wrote the grants and obtained the funding (how this occurred in our context shall be explained in the “The AI MULTIPLY Consortium” section). However, only taking this approach means that other potential venues of influence are missed. Indeed, who the decision-makers are, and what kind of decisions are made are not the same across all projects. Whilst we may typically think of senior team members as decision-makers, everyday decisions are often made by more junior researchers. For example, in interdisciplinary AI research decisions such as what data to include and exclude, model selection, and result interpretation, are often being made every day by Early Career Researchers (ECR) [38–40]. ECRs in this paper are broadly defined in line with UK Research and Innovation as academics who are doctoral, immediately post-doctoral, and/or transitioning to independent research [41]. In this project, in order to impact the ongoing research, ECRs were targeted as they had considerable day-to-day decision-making power.

The benefits of including PPIE voices early include providing time to build up mutual relationships and trust, which have consistently been shown to be vital in PPIE-researcher collaborations [42, 43]. Further to this, early involvement also has the potential to positively impact the ECRs. Undertaking PPIE work can help researchers align their goals with patient-orientated outcomes [44], build research capacity [45], and better communication and planning skills [46]. Further to this, PPIE work is a requirement of many funding grants and engaging early in this process will allow ECRs time to develop these skills and gain experience of translating research concepts for public audiences [47]. ECRs often face structural barriers to undertaking PPIE work, such as the precarious nature of short term contracts [48], institutional constraints to pursuing action orientated participatory work [49], confusion over the flexible nature of the PPIE work [50], and difficulty with communicating complex ideas [50, 51]. Therefore, empowering ECRs to engage with PPIE members requires capacity-building efforts, such as providing the opportunities to communicate effectively with patients and developing their skills in incorporating feedback into their work.

The above has highlighted how current PPIE work in healthcare research may be inadequate if it does not integrate contributor voices into the work, to the right people (those with everyday decision-making power), at the right time. To overcome this, this work is guided by the question: How can PPIE effectively target everyday decision makers in an AI-in-healthcare research project to positively influence the research process? We explore how an NIHR funded AI MULTIPLY project attempted to overcome these issues by initiating the “ECRs meet PPIE” sessions. These sessions encouraged proactive and early involvement of PPIE with those who were making many of the decisions on areas of research such as data inclusion and exclusion, model selection, model training, result interpretation and visualisation. This aimed to democratise the research, meaning public involvement being concerned with people having more say on what impacts them [52]. The methods detail the AI MULTIPLY project, “ECRs meet PPIE” sessions how they worked, and the ethnographic observations. Through observations gathered by the first author, along with surveys filled in by ECRs and PPIE contributors after the sessions, the findings show how contributors felt that their voices were included, we highlight concrete cases where ECRs incorporated the patient voices and show how ECRs learned skills to take forward into future PPIE work. The paper concludes by addressing limitations and provides recommendations for future AI-in-healthcare research projects to help democratise research by targeting everyday decision makers.

## Methods

### The AI MULTIPLY consortium

**Fig. 1:**
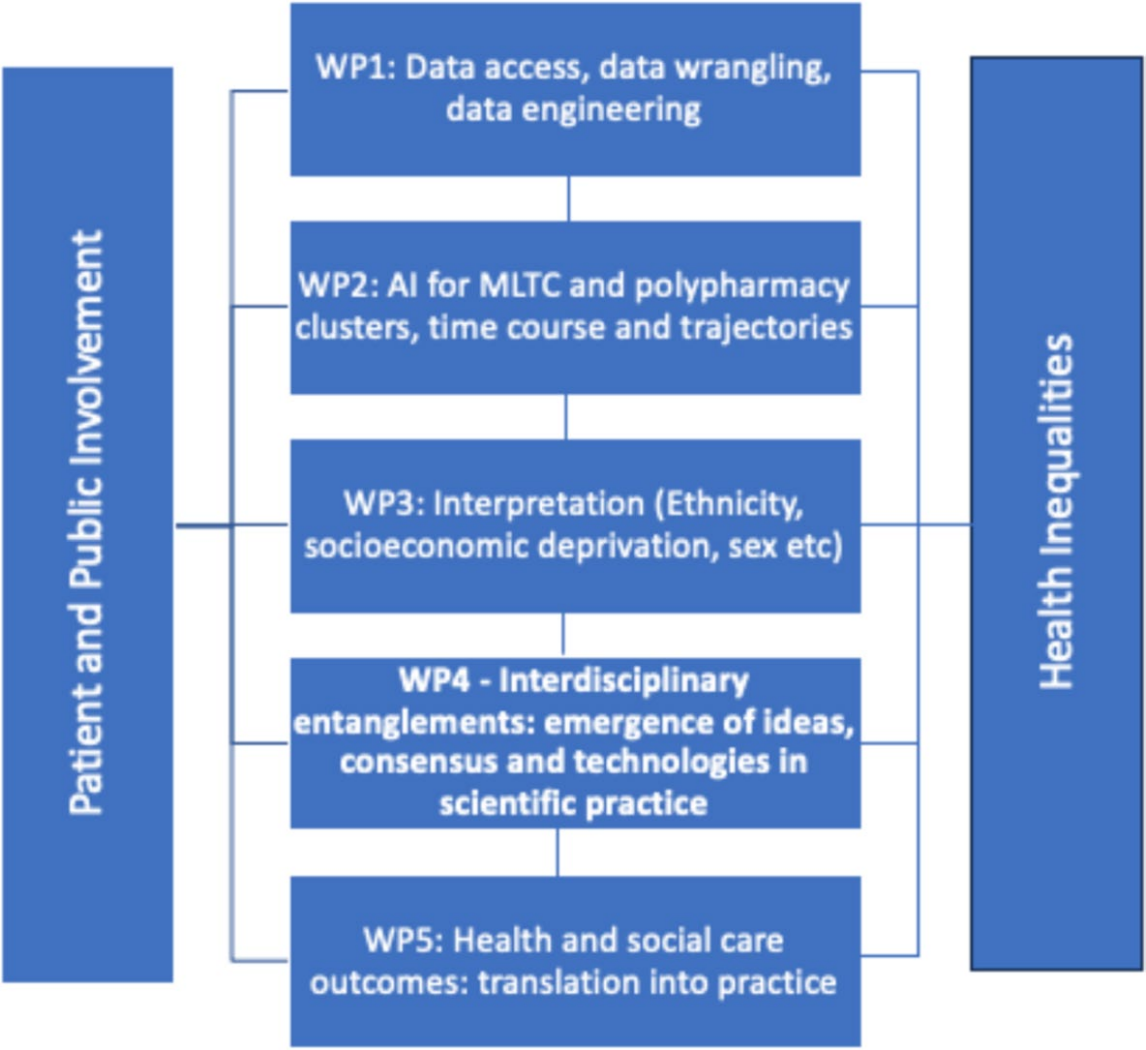
AI Multiply work package structure

This research concerns the AI MULTIPLY consortium. AI MULTIPLY is an NIHR funded, interdisciplinary and multi-method project that aims to use the collective expertise of patients, clinicians, researchers and artificial intelligence to improve the care of people who live with many health conditions and medicines [53]. It is one consortium of the larger Artificial Intelligence for Multiple Long-Term Conditions (Multimorbidity) call, which supports research to use AI and data science to address the challenges of multiple long-term conditions [54–56]. The project team is made up of academic researchers from two large Russell Group Universities in England: Queen Mary, University of London, and Newcastle University. The consortium is split into 5 interdisciplinary work packages (WP), with the two overarching themes of “PPIE” and “Health Inequalities” (Fig. 1). An Independent Advisory Board along with the NIHR Support Facility provide oversight to the project. The PPIE Management Group helped to oversee the PPIE work. Disciplines represented in the team include clinicians, data scientists, data engineers, AI modellers, epidemiologists, pharmacists, and social scientists. Details of each work package can be found in the Appendix B.

The project embedded PPIE from the start. During the development award, AI MULTIPLY engaged with 42 people with experiences of long-term health conditions, social inequalities, and from ethnic minority groups [57]. This engagement was with the senior project members, such as principle and co-investigators, and work package leads. In practice this meant PPIE input at multiple interdisciplinary and work package meetings, as well as management and strategy meetings. Once the project began, PPIE had regular attendees at interdisciplinary meetings, as well as this specific PPIE sessions with the different work packages were established (evaluation of this PPIE involvement is beyond the scope of this paper and shall be written about elsewhere). This helped to shape the overall strategic direction of the research project. However, as shall be discussed, left a gap for influencing everyday decisions.

### The PPIE group

The project has input from 2 PPIE groups, known as the “Newcastle Group” and the “London Group”. This work concerns the Newcastle Group and all references to the PPIE group in this paper refer specifically to work done with them. Recruitment for the PPIE group was overseen by the PPIE management team. Recruitment occurred through use of the existing Newcastle University PPIE networks; VOICE UK and Cumbria, Northumberland, Tyne and Wear NHS Foundation Trust (CNTW) as well as through professional connections of AI MULTIPLY’s Principal Investigators (PIs). The PPIE group initially had 30 members, with a regular core of around 20 and were geographically spread across the UK. The PPIE group is a mix of experienced PPIE contributors, and those for whom AI MULTIPLY is their first experience of research. All members are contributors with lived experience of multiple long-term conditions and taking multiple medicines and/or carers for those living with multiple long-term conditions. For the majority, this was their first experience of working with a health data research project.

The PPIE Group was organised and run by the PPIE Management Team who meet regularly once per month. This was set up to coordinate PPIE activities across the research programme and its various work packages, and in order to have oversight and share learnings across the community. The funding bid highlighted the public involvement to be a key and integral part of the project and due to the unusually large group of PPIE contributors, the number of researchers and WP’s forming the project, a management team was deemed to be optimal in facilitating the PPIE within this project. The management team is made up of two PPIE contributor leads, three other PPIE contributors, one AI MULTIPLY Project Managers, an academic evaluator, and an academic ethnographer. Sometimes, other PPIE contributors, academics, and/or representatives of the London Group were also invited if their opinions and expertise were required. The PPIE contributors on the management team all had lived experience of a range of multiple long-term conditions, as well as diverse experience of being involved with research. The two academics come from different disciplines, with the evaluator coming from a psychology background and the ethnographer from the social sciences. The management team consisted of 6 women and 2 men (self-identified), of which 7 are self-defined white and 1 self-defined south Asian.

### “ECRs meet PPIE"sessions

The"ECRs meet PPIE"sessions emerged as a response to discussions in PPIE management meetings, where talks took place over ways to enhance the influence of PPIE on decision-making within the project. The PPIE group had repeated the message “Remember—If you are making a choice/decision/assumption then you can always ask the PPIE group is this what people actually feel, experience or want?​” many times during full team and management meetings. However, the PPIE management team felt that this message had not been fully taken on board at all levels. Consequently, they recognised the need for a standalone dedicated platform to facilitate direct interaction between PPIE contributors and ECRs, who were understood as the everyday decision makers. The PPIE management team were empowered to design and implement the sessions. Details of this emergence shall be explored in the findings.

The idea received support from the AI MULTIPLY lead investigators and management team and funding was allocated from the initial PPIE budget for the “ECRs meet PPIE” to run. The PPIE Management team took on responsibility for setting up the sessions as it was felt that ECRs may lack the experience or confidence to do so themselves. Structured as twice-monthly 1-h long, online sessions, they encouraged ECRs to share work-in-progress at any stage, accompanied by specific questions for PPIE contributors. This was done to help foster an open environment for the exploration of new ideas. Support was given by the project and work package leads, and they encouraged ECRs to sign up at the weekly data management meetings. The sessions began in May 2024 and at the time of writing, 12 sessions of which 11 were presentations by ECRs, and 1 was introductory have taken place. All work packages have been represented with a large range of topics discussed. An average of 19 PPIE contributors attended each of the sessions. Prior to each session a small description of what would be discussed was sent around. A list of these sessions is in Table 1 below.

**Table 1:** “ECR meets PPIE” session schedule

Session number	Work Package ECR is from	Title of session	Date
1	All	Introduction to “ECRs meet PPIE” sessions	May 2024
2	WP1	Early onset Type 2 Diabetes outcomes and language models	May 2024
3	WP1	Challenges with prescription data	June 2024
4	WP4	Social Science and AI MULTIPLY: understanding how sticking points are negotiated	June 2024
5	WP1	Burst and Train Analysis	July 2024
6	WP3	Experiences of multimorbidity and polypharmacy Qualitative Study: Emerging Themes	July 2024
7	WP5	A study design to emulate a non-inferiority clinical trial of statin deprescribing in older adults using routinely collected primary care data	September 2024
8	WP1	Modelling to identify patterns of multimorbidity and polypharmacy	September 2024
9	WP2	Surveying language models for health outcome prediction using transfer learning	October 2024
10	WP2	Predicting treatment outcomes in psoriasis using registry data	October 2024
11	WP2	Predicting Chronic Kidney Disease Progression using Language Models	November 2024
12	WP4	What should be"explainable"about AI?	November 2024

### Understanding the sessions

The sessions were observed ethnographically [58] by the first author as part of a wider ethnographic study on the interdisciplinary team workings of the AI MULTIPLY consortium. To date, the first author has conducted over 220 h of participant observation [59] across the AI MULTIPLY project. The lead ethnographer participated as a member of WP4. Observations took place in person and online and covered: work package meetings, interdisciplinary team meetings, management meetings, steering group meetings, reflexive interdisciplinary workshops, PPIE management meetings, and a range of PPIE sessions. For this paper, only observations from PPIE management meetings and PPIE sessions are within scope. Detailed fieldnotes were written the same day, inspired by Emerson, Fretz & Shaw’s taking of descriptive notes plus analytic memos together [60]. This formed the first stage of analysis. Data was analysed inductively from fieldnotes. All the PPIE management meetings and sessions were recorded (with consent) and these recordings were used to support accuracy during the data. Analysed data was shared with the whole authorship group in the form of ethnographic accounts for analysis, reflexion, and comment. Unconscious bias was addressed through continuous reflexive practice. The first author ethnographer kept a reflexive diary as part of fieldwork collection [61] and fieldnote data was regularly discussed and shared with other team members. When points of accuracy arose, the meeting recordings were checked.

Additionally, the PPIE group conducted an evaluation for internal use so that they could track what was going well, and what need to be adapted to ensure continued smooth running. The survey method was selected by the PPIE management team, with these being designed and circulated by the second author. Surveys were perceived to be an effective way to capture the opinions and experiences of both ECRs and PPIE members involved in the sessions [62–64], whilst reducing administrative burden of other types of feedback such as interviews. The surveys varied slightly between the groups to capture the different experiences of presenting and contributing. The surveys were sent to all involved in the sessions within 24 h of their taking place, to help ensure that what had taken place was fresh in everyone’s minds. The surveys were designed for an internal audience, and details of each can be seen in Appendix C. Overall, 11 ECR and 31 PPIE contributor surveys were completed. The insights from these surveys shall be woven into the ethnographic accounts of the sessions given in the findings below. High-level results from the quantifiable sections of the surveys can be seen in Appendix D. These survey results were analysed alongside the ethnographic fieldnotes to expand the findings by incorporating the perspectives of the ECR researchers and PPIE contributors of the sessions [65]. Whilst the ethnographic data provide the basis for the findings, the surveys provided important information not gathered through the observations, such as what changes AI modellers made to their work post sessions.

### Findings

The findings show an account of how the “ECRs meet PPIE” sessions came about, followed an ethnographic case study of two of the sessions to show how they ran and were received. Where names are used, they have been pseudonymised. Sessions 9 and 10 were selected by the first author as the ethnographic accounts presented here due to the range of discussions within them, as well as the positive and challenging aspects of their running. Session 9 with Hasan has been regularly referred to as an example of positive PPIE-research collaboration, with complex ideas being explained in multiple ways and concrete changes to the AI work implemented afterwards. Session 10 with Hope highlights some of the challenging aspects of the sessions, which led to changes to the ECR meet PPIE format by the management team for the sessions moving forward.

### An ethnographic account of forming of the “ECRs meet PPIE” sessions

The monthly PPIE Management Meeting took place online as usual, to accommodate the fact that the people who sat on it were located across the UK. Whilst normal meeting formalities such as agendas were always sent out in advance, these meetings had a relaxed air to them, which fostered a jovial and inquisitive atmosphere. Even when things “got down to business”, it was not uncommon for there to be jokey and lighthearted interjections, often related to the lives of those who were present. The whole management team were present at this meeting. This particular meeting took place about a third of the way into the project funding timeline. At this meeting were the regular eight members of the PPIE management team plus a PhD candidate who had helped facilitate PPIE training earlier in the project.

The main issue on the agenda for this meeting was to reflect on the recent PPIE sessions organised with Work Packages 1 and 2. Whilst the discussion started positively with how the work package researchers had been very welcoming to the patient group, and had listened and answered questions well, it became clear that there was a growing concern about the alignment between PPIE input and research decision-making. A particular example was raised from the meeting with WP1. Here, WP1 had presented what healthcare data they had available for the project. The data came from two main sources, UK BioBank and the Clinical Practice Research Datalink (CPRD). When explaining how the team were going to use this data to train their AI models to predict outcomes such as mortality and time to next disease, the discussion shifted to what could be seen in the data that the patients felt would be more beneficial for them, mainly “quality of life” and “periods of wellness” (as opposed to periods of sickness). While these priorities resonated strongly with the PPIE contributors, they had little traction with the researchers'work because such concepts were not directly observable in the available data. From this moment on, the session had reached an impasse.

Reflecting on what had worked and not worked led one PPIE contributor to talk about their experience as a representative on the AI MULTIPLY weekly “data engineering” meetings. By attending these weekly meetings, it was hoped that PPIE would have a voice during technical conversations concerning the data. However, the PPIE contributor said that the meetings don’t generally have an agenda in advance and the meetings constituted a continuing dialogue on several parallel projects that are ongoing. Two public contributors were involved in these meetings but each of them is only able to attend one meeting per fortnight (due to budgetary and time constraints) and it is not possible to tell which meetings are the most important to attend. As a result of this, the patient voice was seen as intermittent, and the dialog somewhat punctuated. It was noted that this was a departure from the grant writing phase of the project, when PPIE involvement had been more consistent.

Talking about this promoted a broader discussion in the PPIE management meeting about where decisions were actually being made in the project, and how the PPIE contributor voice could better be targeted to benefit the research. It was here that the PhD researcher spoke of their experience of working on the project. They said that because the project was big data/AI, then ECRs (including themselves) were constantly making micro decisions. They gave examples of their choosing which type of AI model to use, and how to interpret the results. As a result of this, they speculated that if there was a way that ECRs could be specifically targeted, without higher ups present, then PPIE contributors’ voices could then be heard. They concluded that unlike WP leads, who shaped overarching strategy and had been targeted previously, ECRs engaged in the micro-decisions that determined the trajectory of the research often without people really knowing. Sometimes the micro-decisions they made early in a project would become apparent much later, and when questioned were re-visited resulting in the need to reprocess and reanalyse the data.

The meeting discussion continued, and it was agreed that if future PPIE sessions were designed to engage ECRs directly, rather than primarily senior leads, the likelihood of PPIE input shaping the research would increase. One PPIE contributor added in that from their PPIE experience they could also see two-fold benefits to targeting ECRs. First, the PPIE voice could be better integrated into the project at a stage where methodological decisions were still fluid. Second, it was hoped that ECRs would develop PPIE engagement skills, a capacity that would serve them in their future careers as PPIE work is increasingly becoming a requirement of research funding.

The discussion concluded with agreement on the creation of “ECRs meet PPIE” sessions. These would be set up by the PPIE management team so that ECRs could come and discuss work in progress with contributors. There was broad agreement that these sessions would be standalone, where work-in-progress would be shared, and that ECRs should also come with specific questions which they felt PPIE contributors could help with (even if these were not stuck to), so that the sessions did not turn into simply telling but discussing. These sessions would take place alongside the already planned PPIE work with each work package. The rationale being that PPIE contributors did not have to attend all of the sessions to build up knowledge (although they were welcome to), but that each session would be on a specific problem/issue that the ECRs and PPIE contributors could collaborate on together. The meeting ended with people optimistic for this new direction and excited to see what would come moving forward, but also a recognition of the ongoing challenges of translating engagement into impact.

After the meeting work began on setting up the sessions. They were announced to the research team, and a link was sent around to the ECRs to sign up. However, there was initial hesitation from the ECRs, perhaps due to uncertainty over what the sessions entailed and their lack of experience of engaging with PPIE contributors. It was decided that at first a welcome session would be organised so that the ECRs could learn about the rationale for the meetings and how they could be supported through them (for example members of the PPIE management team reached out to ECR to offer help crafting their sessions if they wished for it). This seemed to help as some ECRs signed up, however not all. The PPIE group had to work by sending targeted emails to specific ECRs to get them registered and ask for help from the PIs to encourage their ECRs to participate. These prompts had the desired effect, with some ECRs signing up to multiple sessions to gain PPIE involvement on different aspects of their work.

### An ethnographic account of “ECRs meet PPIE” session 9 – Hassan

The meeting began just before 9am on a Wednesday morning. Originally, there had been plans for it to happen on a Friday, but various members of the public and research team had pointed out that this would exclude Muslim members from attending who were attending prayers. It was also acknowledged that 9am would not work for everyone, for example those doing the school run. Based on the survey feedback of previous sessions, a plan was put forward to alternate times and days of these sessions moving forward.

The session opened with an update from Helen, the PPIE contributor who chairs the sessions, that she had received from Kenji, the ECR who presented 2 weeks prior. Helen said that Kenji had been in touch to thank everyone for their contributions and that additional lifestyle factors, especially those around heavy alcohol use and smoking were now being incorporated into his work as a direct response to the discussions during his session. Jennifer, a PPIE contributor, interjected with a slight regret: “unfortunately, I wasn’t at the last meeting” highlighting both the challenge of continuity in PPIE engagement, but also how engaged the wider group were with the “ECRs meet PPIE” format to date. However, these sessions were designed to be standalone, so Jennifer not attending the previous meeting did not have an impact on her ability to contribute to Hassan’s.

The focus then shifted to Hassan, the ECR talking today. Hassan is an AI modeller and is presenting his work on leveraging Large Language Models (LLMs) for predicting different healthcare outcomes. He had been prompted to sign up through a targeted email from the Helen after she noticed he was not registered to give a session. Before beginning in earnest, Hassan set a clear intention that he hoped that not only would he get useful input on his research, but also that “it will be very reassuring if you can understand what I say today even if you don’t know what a large language model is, or the specifics of the AI models”. For Hassan, success of the session would not only be benefits to his research, but he also wanted to be educational in what he said to the group.

Breaking down his research, Hassan explained how he was utilising existing LLMs rather than constructing new models from scratch. As he was giving an example of feeding patient medication histories into pre-trained models to explore predictive power, Flora (a PPIE contributor) interrupted with a pointed question; “we had a previous speaker who told us about the probabilistic nature of LLMs, but what they didn’t explain, and so far, you haven’t either, is what’s the context? How do you go from a prompt to predicting the next word? Because you have to start from somewhere. What is that process?”. The previous speaker Flora was referring to was Angela, who had presented at the second “ECRs meet PPIE” session on her use of LLMs. Whilst most of her session had gone smoothly, there had been some confusion over her description of what an LLM was. She had asked contributors to think of them as “very smart predictive text”, however this had not resonated with all the contributors who saw LLMs such as ChatGPT as answering a question, rather than predicting a next word. Attempts to use different metaphors and similes by Angela worked for some but not for all contributors.

Hassan seemed to recognise from the question that Flora had some knowledge (perhaps from previous sessions) on how AI and LLMs worked, and gave a reply focusing on prompt engineering, how models are robust to variations in phrasing and stating that models can recognise overarching themes well without getting bogged down in specifics. Flora responded warmly with “that was wonderful, thank you, thank you so much”. When Hassan moved on from this, he appeared to be aware that the answer he gave might not have been appropriate for everyone, so when justifying his use of LLM selection used a simpler analogy of riding a bike; “When you ride a bike as a kid, you have training wheels. The large model acts as training wheels for the smaller models.” Here, Louisa, a PPIE contributor, interjected appreciatively: “I love the way you explain stuff.” This range of examples to help explain was picked up on in a PPIE feedback survey on Hassan’s session, which said that the “learning to ride a bike analogy was much more useful than [other] examples”. However, it also highlighted the difficulty in explaining complex terms around AI, as both Hassan and Angela before had difficulty in picking the correct examples and metaphors to use to get their points across in an understandable manner.

A series of questions were asked by contributors when Hassan showed a slide of his findings so far. Lorenzo queried the comparative advantage of Hassan’s LLM approach over existing risk scores. Hassan acknowledged the question’s importance and distinguished his method as more individualised than traditional population-level risk assessments. He also recognised AI’s persistent biases, albeit with an optimism that careful methodology could mitigate them. In his post-session survey, Hassan wrote that he had taken onboard the comments on risk scores and said that he will use these scores as a baseline against which to test his models moving forward, showing a direct impact on the research from the session.

The conversation turned pragmatic when Shirely inquired about data anonymity. Hassan assured that all the data which was being used on the AI MULTIPLY project was anonymised upon receipt. However, he admitted that “there might be an ethical red flag because the models we are using are open source, but they have already been filtered by Microsoft and Google.” Here, the concern was beyond the scope of what could be done by Hassan, and beyond his immediate scope of knowledge. This highlighted that whilst the “ECRs meet PPIE” sessions were a good forum for many discussions, in a similar way to the old PPIE methods, some things were simply beyond the scope of what was possible.

As Hassan moved through his results, he talked through dataset structuring and model evaluation, using familiar descriptions such as barcodes to explain complex and technical elements such as “embeddings”. During this explanation, Lorenzo inquired about the definition of “multimorbidity” he was using for his model that predicted what disease a patient would next have. Hassan was candid and said, “I am going to be honest; I have listened to the clinicians on the team for these definitions.” This highlighted the complex nature of an AI-in-health project such as AI MULTIPLY. Whilst ECRs were making lots of everyday micro decisions, they also relied on other team members from other disciplines to fill the knowledge gaps that they had. In this case, Hassan, an AI modeller, was relying on clinicians for medical definitions, and therefore he was not able to respond or adapt to patient comments.

The meeting ended on an encouraging note, with multiple participants expressing appreciation for Hassan’s ability to simplify complex concepts which was also highlighted in their feedback forms. Many said the terminology was explained well, and they felt that questions were welcomed and answered well. As Hassan reflected in his own survey feedback, the PPIE group challenged him to think about alternative implementations and reinforced the societal importance of his work. Hassan also made concrete changes to his work based on the session, where he added benchmarking his model against existing risk scores, as queried by Lorenzo. This feedback was reflective of many of the other ECRs, who all said that they had found the sessions useful with 8 out of the 11 ECR surveys saying the sessions would “definitely” influence their work, and the remaining 3 stating it was “possible” that it would influence their work. For example, the ECR survey from session 11 said how they had changed their analysis of chronic kidney disease (CKD) in light of what had been spoken about by contributors on age-related CKD and pathological CKD. They also spoke about scheduling a further meeting to help focus their work on “patient-centric outcomes”. Further to this, ECR 7 showed how everyday decisions were influenced in terms of which features to include in his work. After his ECR meet PPIE session he included more lifestyle factors based on feedback he had received. On Hassan’s session, feedback from the PPIE contributors was also positive, but some highlighted how they did not speak up because others with seemingly more knowledge on the topic were more vocal. The issue of certain people dominating the conversation was also raised in feedback from Angela from her session, and the PPIE management team promised to discuss what could be done to hopefully encourage everyone to share their view and to reduce domination by a few people.

### An ethnographic account of “ECRs meet PPIE” session 10 – Hope

The virtual Zoom meeting room flickered to life as participants joined, their screens filling the grid with different faces from patients and the facilitator. Hope, a clinician-turned-PhD researcher joined ready to present her work on using AI to personalise biologic treatments for psoriasis.

Helen started by saying could Hope open with questions she would like answered, so that the discussion can have some focus. Hope said she wanted opinions about the utility of the research and suggestions around psoriasis and how to research it. Hope herself noted that this was “fairly vague”. Hope also encouraged questions throughout the session, as she would prefer to be interrupted than people save questions for the end, so that discussion can happen in the moment, rather than having to wait and go back at the end.

Hope began her presentation with slides showing a concise overview of psoriasis as a chronic, immune-mediated condition affecting skin, cardiovascular health, and mental well-being. This included some large photographs of some more extreme cases of the disease. This then led her to highlight the promise but also limitations of current biologic therapies. While they may be beneficial for some, they can fail to control the disease or cause side effects for others which in the UK means around 25% of patients change biologic medication within a year of starting. The current prescribing practice, she explained, was largely trial and error which is a burden for both clinicians and patients. Her central research question emerged from this challenge: Can AI help personalise biologic prescriptions to improve patient outcomes. In other words, can AI help the clinicians to pick the best biologic for a patient first time?

At this point, around 10 min into the session, Lorenzo raised his hand to ask about the association between psoriasis and cardiovascular disease. Hope acknowledged the complexity, explaining that while the link was established the causality often remained unclear. Jennifer followed, wanting to know more about the role of environmental factors. This was driven by her personal experience of biologic treatment and how her living conditions seemed to influence the medication’s efficacy. Hope admitted that while factors like alcohol, smoking, and BMI were considered, others were not. In the chat, other participants asked about the differences in rural and urban living. Hope mused that this was not something she had considered but if the data available to her included postcodes she could perhaps pursue it as avenue to go down. These opening two questions led to a string of hands being raised, and comments being made in the chat.

MJ’s questions turned to disease presentation across different skin types. Hope explained that variability did exist, highlighting how dermatological conditions often manifest differently depending on skin pigmentation. Flora then asked about causal pathways of psoriasis, which led Hope to discuss known immune pathways targeted by biologics, while emphasising the uncertainty around which pathway drives disease progression in any given individual.

The session had now unfolded into less a structured presentation and more as an open forum for a range of questions and queries. The majority of which seemed to be around psoriasis as a disease, as opposed to Hope’s research specifically. Participants shared personal anecdotes, asked for clarifications, and at times, steered the conversation away from a focus on the research itself. Interspersed between the hands raised, Helen relayed questions from the chat. Hope engaged each question thoughtfully and did not seem to mind that they were often beyond the scope of her research. Questions had now been asked for over 40 min. Hope was still on her research question slide, and I wonder what else she had planned to show the group to gain feedback on. It now seemed past the point where the assembly would return to the presentation, and instead a more open question and answer session had emerged.

Lorenzo raised his hand and asked what appeared to be a more pointed question about the research itself. He asked Hope whether patients wanted personalised biologics, as questions he had found online from patients about psoriasis rarely mentioned this. Hope reassured Lorenzo that she had engaged in other PPIE sessions where her research question had been supported. Lorenzo followed up by asking whether it was appropriate to use AI for this, given how he feared it may negatively impact the doctor-patient relationship. Hope took this on board and assured Lorenzo that her plan for this research was for the AI to be an additional piece of information for clinicians to use when decision making, rather than creating an AI which would be the decision maker itself. After a few more questions from those present, the clocked ticked over the hour mark, and Helen drew the session to a close. The contributors expressed their thanks to Hope and Hope to them.

Reflecting in her survey after the session, Hope expressed gratitude for the diverse perspectives and questions and the opportunity it had given her to develop her communication skills. This development of skills was a common theme across the ECR feedback forms, for example ECR 3 reflected in his survey that his session had made him think about how to strike a balance between informal and formal language when discussing scientific concepts. Similarly, ECR 2 said that they had gained skills on how to navigate the challenges of certain people dominating the floor. However, Hope did note that the session did not lead to immediate changes in her methodology, but it had sharpened her awareness of contextual factors that *could* inform the everyday decisions she made around her future analyses. Participants, too, were divided in their feedback. Some, such as Tim, felt sidelined from contributing to the research agenda because the discussion never got into what work Hope was planning on doing, while others valued the opportunity to discuss their experiences of, and gain more knowledge around, psoriasis as a disease. Ultimately, the session perhaps underscored a core tension in patient and public involvement at large; balancing the open-endedness of democratic dialogue with the specificity of desired actionable research insights.

After this session, Helen and the rest of the PPIE management team had a chat about the feedback in light of the session. It was agreed that having around 20 PPIE contributors attend each session was great in terms of enthusiasm, but perhaps not in terms of discussion if some people felt sidelined and the conversation went off track so as not to be useful for the researchers. A decision was made to cap PPIE contributors at 10 per session moving forward.

## Discussion

The “ECRs meet PPIE” sessions aimed to include patient contributor voices in AI-in-healthcare research by targeting those making everyday decision makers. They came about due to concerns that PPIE interventions were not being as effective as they could be. Further to this, they helped provide these decision makers with the skills and confidence to effectively integrate PPIE into their work. Both of these are important areas as previous research has shown how PPIE in health and care research can occur at inopportune times, and be ineffective [25–28], and ECRs often have limited exposure to PPIE work [44–46]. The findings from the evaluations of the sessions show examples of these goals being met, such as ECRs gaining communication skills, and direct cases of features and outcomes being changed in AI models as a result of the sessions.

The sessions successfully elevated the role of PPIE contributors from a traditionally reactive position, such as reviewing outputs [66], to a proactive role in shaping research directions. Contributors felt that their voices were heard, with a large majority reporting that their input was valued and taken into account. The ECRs reported concrete examples of the influence the sessions had on their work, such as; shaping decisions on data inclusion, encouraging sensitivity analyses for lifestyle factors, and inspiring the creation of patient-centred research outputs. This shift highlights the potential for PPIE to contribute to decision-making when included at earlier stages of research, and by targeting the people responsible for decisions. For the ECRs, the sessions also provided a valuable platform to develop their understanding of PPIE’s importance and its practical application. Researchers reported an increased awareness of diverse perspectives and improved skills in communicating complex ideas to non-technical audiences. These outcomes demonstrate that the"ECRs meet PPIE"sessions not only bridged gaps between patients and researchers but also fostered a culture of collaboration that is essential for advancing patient-centred AI development research in healthcare [11, 17]. The"ECRs meet PPIE"sessions offer valuable insights into the broader challenges and opportunities in democratising healthcare research, particularly in the context of AI in healthcare. By fostering collaboration between patients, the public, and researchers, these sessions address several pressing issues, such as advancing patient-centric AI in healthcare, building trust, and shaping research culture.

A recurring criticism of AI in healthcare is its tendency to develop solutions in isolation from the lived experiences of patients [9, 10]. The sessions demonstrated how early involvement of PPIE contributors with ECRs can help align research directions with patient priorities. By providing feedback on data inclusion, research assumptions, and patient-centred outcomes, PPIE contributors directly influenced the development of more inclusive and relevant AI tools. This approach may help to mitigate some risks such as algorithmic bias, lack of trust, and poor adoption, which are critical barriers to the successful implementation of AI in healthcare [11, 14, 67]. As previous PPIE research has shown, the integration of PPIE at an early stage also has the potential to strengthen trust between researchers and the public, addressing concerns about the opaque and potentially inequitable nature of AI systems [17]. During the sessions, contributors felt that their voices were genuinely heard, with potential to help trust building in new AI systems.

Empowering ECRs with skills in PPIE not only benefits individual projects but also contributes to a broader cultural shift in healthcare research [19]. By beginning to equip the next generation of researchers with the skills and tools to engage with patients and the public, these sessions embedded patient-centred practices into the fabric of the AI MULTIPLY project. As ECRs advance in their careers, the lessons learned from these sessions have the potential to influence the wider research landscape, fostering a more collaborative and inclusive approach to innovation.

### Challenges and limitations

However, there were tensions and some difficulties concerning the sessions. The “ECRs meet PPIE” sessions began nearly halfway through the funding time of AI MULTIPLY. By this point, many project decisions had already been made on important areas such as AI model prediction outcomes. Some (but not all) of these decisions had either been made without PPIE representation or had superficial engagement. In future, it would be advisable for future projects to set up these sessions at an earlier point in the project. A tension which emerged from the sessions was that between rights based and value (democratic) based values of PPIE research [52, 68]. The sessions were set up with the aim of including more PPIE voices in the on-going research and saw value in both rights and value-based approaches. Hassan also highlighted that he wished for both impact on his research, but also for a more democratic inclusion of a range of people in the research process. The tension was keenly felt in the reaction to Hope’s session where many contributors were happy to be included and have a forum to ask general questions, whereas others were left frustrated that there was little concrete impact on the research. These tensions must be recognised and understood to help make PPIE involvement beneficial to all. This led to changes to the running of the sessions where the number of participants was capped at 10. Whilst helping to avoid the problem of too many people, it opened up other problems of excluding PPIE contributors who wanted to attend from attending or requiring ECRs to run the same session twice with different groups of patients and thereby increasing their workload. As well as this, other known difficulties also emerged such as explaining complex topics to lay audiences. As seen in the ethnographic account of Hassan’s session, despite using multiple metaphors and ways of explaining technical AI language, there can still be problems translating these concepts to patient groups [51, 69, 70]. We also note that the racial make-up of the PPIE management team is not very ethnically diverse. This is a well document problem with PPIE work [71] and something that has been reflected on many times during PPIE management meetings. However, we maintain that in terms of lived experience of multimorbidity, and of healthcare data research, there is a high degree of diversity.

### Future guidance

We have been guided by the questions “How can PPIE effectively target everyday decision makers in an AI-in-healthcare research project to positively influence the research process?” and have provided evidence of the success of “ECRs meet PPIE” sessions. From our work, we recommend the following for those who wish to set up a similar initiative:

1. Identify which members of the research team are making everyday decisions.
  For AI MULTIPLY, this was ECRs engaging in data work, but this may not be the case in other settings. Therefore, those who are making everyday decisions need to be identified so that opportunities for engagement are not lost. In the findings, we showed how research decisions were directly impacted by this work.
2. Have a clear structure in place and secure senior support.
  The sessions in the AI MULTIPLY project were twice monthly and had the support of the project and work package leads. This was important during difficulties of signing up ECRs, as PIs were able and willing to use their influence to encourage engagement.
3. Establish a culture where those presenting felt empowered to present work-in-progress, and where PPIE members were encouraged to share insights candidly.
  It was normalised to share early stages and unfinished work (as seen in the findings). This was important to allow early involvement from PPIE. Further to this, support was provided from experienced PPIE members so that researchers could have a point of access if they have questions or concerns before and/or after their sessions. As many of the targeted researchers were new to PPIE work on AI MULTIPLY, this helped mitigate some of the apprehension around engaging with a new type of work.
  Normalise sharing of unfinished code and analyses.
4. Allocate appropriate resources for the sessions to take place.
  This includes finances to renumerate contributors for their time, and admin support to help with the setting up of meetings, answering of emails, and experienced facilitation for the meetings to support the ECRs to develop these skills. This important for all PPIE but is often overlooked.
5. Be flexible based on constant feedback.
  People’s lives and circumstances can change, and flexibility to allow for a range of contribution is necessary. In the case of AI MULTIPLY, sessions were originally planned on Fridays, but due to feedback from ECRs and contributors that this was inappropriate for Muslim members of the team as they would be at prayers, the sessions were moved.

## Conclusion

The"ECRs meet PPIE"sessions represent an innovative approach to addressing two critical challenges in healthcare AI research: increasing the influence of PPIE contributors in shaping research outcomes and equipping ECRs with essential skills to integrate PPIE into their work. By creating a space for dialogue and collaboration, these sessions have demonstrated their potential to transform both research practices and outcomes. For PPIE contributors, the sessions moved beyond tokenistic engagement, empowering them to play an active role in decision-making processes and ensuring their voices influenced foundational research elements. For ECRs, the initiative provided a unique opportunity to refine their communication and collaboration skills, helping them understand and incorporate diverse patient perspectives into their work. The dual impact of the sessions highlights their value as a tool for democratising healthcare research and fostering patient-centred innovation [62].

In a rapidly evolving field like healthcare AI, it is imperative to ensure that technological advancements are both patient-centred and socially responsible [11]. The"ECRs meet PPIE"sessions exemplify how collaboration can bridge the gap between researchers and the public, offering a path forward for more democratic and impactful innovation in healthcare research. The broader implications of this work point to a future where patient-centred research is the norm rather than the exception.

## Supplementary Information

Supplementary Material 1.

## Figures and Tables

**Figure:**
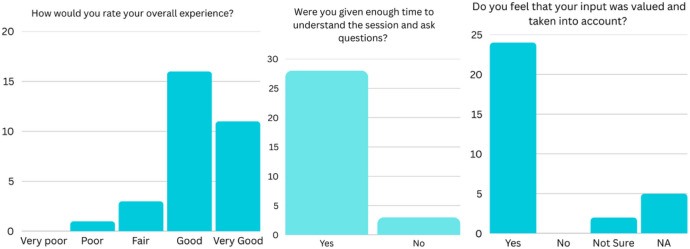
The vast majority felt that the sessions were “good” or “very good”, with only 1 stating they were “poor”. Contributors overwhelming felt that they were given time to understand and ask questions, and that their input would be taken into account

**Figure:**
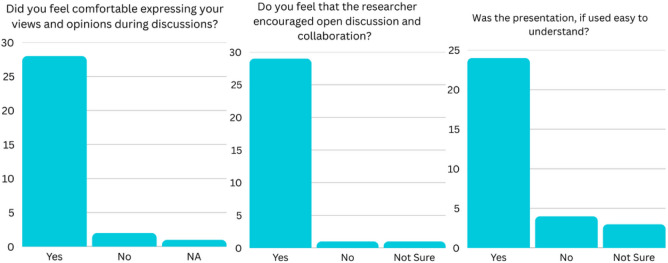
Contributors also overwhelmingly felt comfortable expressing views during the sessions, and that the researchers encouraged open discussion. Presentations from the ECRs were also on the whole seen as easy to understand

**Figure:**
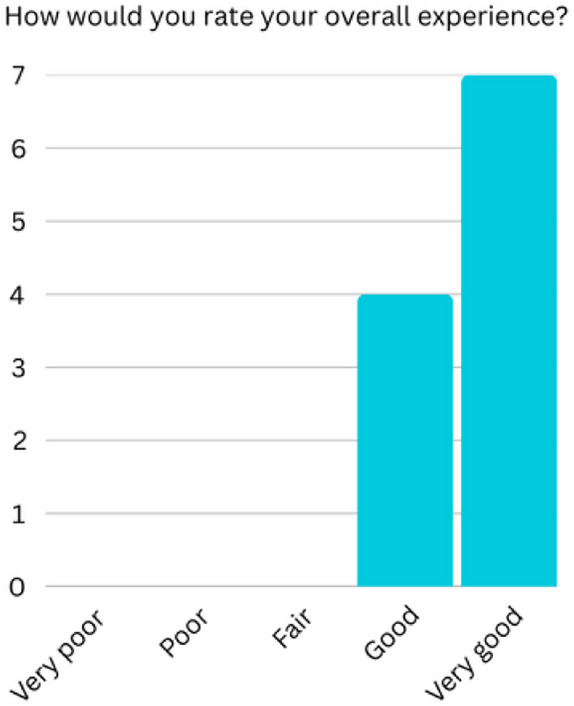
ECRs reported similarly positive experiences of the sessions, with all rating them “good” or “very good”

**Figure:**
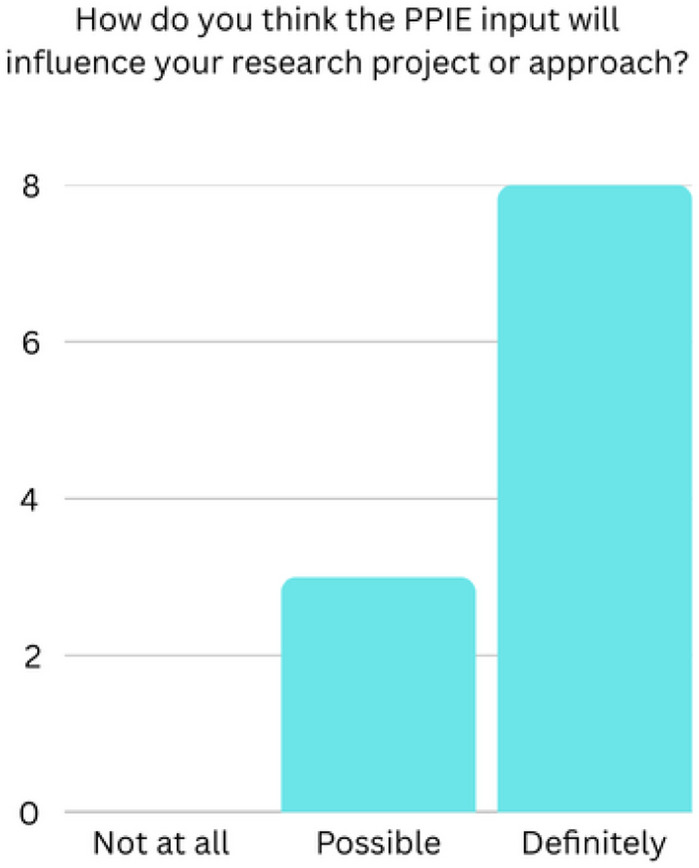
Out of the 11 ECRs, 8 said that the PPIE meets ECR session would “definitely” have an impact on their work, and 3 said it was “possible”. None stated that there would be no impact

## Data Availability

No datasets were generated or analysed during the current study.

## Appendices

**Appendix A - AI MULTIPLY Consortium**

Vanesa Apea (Barts Health NHS Trust) Mike Barnes (Queen Mary University of London, joint PI) Victoria Bartle (Patient and Public group representatives, AI MULTIPLY Consortium, UK) Alastair Burt (University of Newcastle upon Tyne) Dexter Canoy (University of Newcastle upon Tyne) Megan Clinch (Queen Mary University of London) Ceri Durham (Patient and Public group representatives, AI MULTIPLY Consortium, UK) Sarah Finer (Queen Mary University of London) Olivia Grant (Patient and Public group representatives, AI MULTIPLY Consortium, UK) Soraia Guerra-Sousa (University of Newcastle upon Tyne) Barbara Hanratty (University of Newcastle upon Tyne) John Isaacs (University of Newcastle upon Tyne) Tom Lawton (Bradford Teaching Hospitals NHS Foundation Trust) Hamish McAllister-Williams (University of Newcastle upon Tyne) Paolo Missier (University of Newcastle upon Tyne) Chris Plummer (The Newcastle upon Tyne Hospitals NHS Foundation Trust) Nick Reynolds (University of Newcastle upon Tyne, joint PI) Sohan Seth (University of Edinburgh) Deborah Swinglehurst (Queen Mary University of London) Adam Todd (University of Newcastle upon Tyne) Neil Watson (The Newcastle upon Tyne Hospitals NHS Foundation Trust) James Wason (University of Newcastle upon Tyne).

**Appendix B**

**Table 2:** The Work Packages

**WP1: Data access, data wrangling, data engineering**	Data from regional and national electronic health records were acquired, cleaned, and engineered for use later in the project.
**WP2: AI for MLTC and polypharmacy clusters, time course and trajectories**	AI techniques are used to analyse the data prepared by WP1. WP2 aim to discover relationships between MLTC, polypharmacy and personal/social factors. These are then repeated across different datasets to compare the results to findings in similar studies. In practice, work packages 1 and 2 work very closely together and are often indistinguishable.
**WP3: Interpretation (Ethnicity, socioeconomic deprivation, sex etc)**	Looks at how the results from WPs 1 and 2 can be applied to real-world situations. The research here is divided into three parts: mental health, inflammation, and how ethnicity and social factors impact treatment options.
**WP4 - Interdisciplinary entanglements: emergence of ideas, consensus and technologies in scientific practice**	WP4 is the social science package and aims to explore how all the different people in the AI MULTIPLY collaboration work together in practice and how this informs the design of the Artificial Intelligence technology to understand how successful collaborations can be achieved in the field of Artificial Intelligence in healthcare.
**WP5: Health and social care outcomes: translation into practice**	Research from other parts of the project will identify which people are more likely to experience harm or side effects from their medicines. This WP is focused on making sure these findings can be used in practice to help improve the treatment of people with different long-term conditions. This is achieved through trial emulation.
**Overarching theme 1: Patient and Public Involvement **	PPI is an integral part of our project and is woven through all elements, including design (addressing underserved groups – see below), conduct (a dedicated Patient Advisory Group informing the management committee), and dissemination plans.
**Overarching theme 2: Health Inequalities**	An understanding of health inequalities is designed to be built into all stages of the research project. The knowledge of inequalities is used to understand when best to intervene to reduce health disparities between patient groups and improve outcomes.

**Appendix C**

**Table 3:** The Surveys

**ECR Survey Questions**	**PPIE Contributor Survey Questions**
How would you describe your overall experience of the PPIE meeting. (Very good, good, unsure, poor, very poor)	How would you rate your overall experience with this session? (Very good, good, unsure, poor, very poor)
How do you think the PPIE input will influence your research project or approach? (not at all, possible, definitely)	Were there any challenges or negative aspects you encountered?
Were there any concrete changes to your research	Were you given enough time to understand the session and ask questions?
Were there any new insights or perspectives from the PPIE group that you hadn’t considered before? Yes/No (please expand)	Do you feel that your input was valued and taken into account?
Did the feedback from the group challenge or change your assumptions Yes/No expand if possible	Did you feel comfortable expressing your views and opinions during discussions?
What did you learn about the importance of PPIE in research from this meeting?	Do you feel that the researcher encouraged open discussion and collaboration?
What skills or knowledge do you feel you’ve gained from participating in this meeting.	Was the presentation, if used easy to understand?
Did you feel adequately prepared for the PPIE meeting? If not, what could have helped?	How do you think your involvement contributed to the research?
Further comments	Do you believe that the PPIE activity will lead to positive outcomes or changes?
	Was there any terminology and not explain?
	Further comments

**Appendix D**

**1 PPIE experiences of the sessions**

The experience of the PPIE contributors to the sessions was very positive, as seen in the tables below.

**2 Impact of PPIE sessions on ECRs**
