# Supplementary material for: Targeting everyday decision makers in research: early career researcher and patient and public involvement and engagement collaboration in an AI-in-healthcare project
Source: Res Involv Engagem. 2025 Aug 19;11:100. doi: 10.1186/s40900-025-00753-9 (PMC12366416; doi:10.1186/s40900-025-00753-9)
Supplement: Supplementary file 1 — Supplementary Material 1. [file 40900_2025_753_MOESM1_ESM.docx]

| **Section and topic** | **Item** | **Reported on page No** |
| --- | --- | --- |
| 1: Aim | Report the aim of PPI in the study | P3-4 |
| 2: Methods | Provide a clear description of the methods used for PPI in the study | P4-7 |
| 3: Study results | Outcomes—Report the results of PPI in the study, including both positive and negative outcomes | P7-14 |
| 4: Discussion and conclusions | Outcomes—Comment on the extent to which PPI influenced the study overall. Describe positive and negative effects | P14-16 |
| 5: Reflections/critical perspective | Comment critically on the study, reflecting on the things that went well and those that did not, so others can learn from this experience | P14-16 |
